# Docetaxel (Taxotere) is active in non-small-cell lung cancer: a phase II trial of the EORTC Early Clinical Trials Group (ECTG)

**DOI:** 10.1038/bjc.1994.311

**Published:** 1994-08

**Authors:** T. Cerny, S. Kaplan, N. Pavlidis, P. Schöffski, R. Epelbaum, J. van Meerbeek, J. Wanders, H. R. Franklin, S. Kaye

**Affiliations:** Institut für Medizinische Onkologie, Berne, Switzerland.

## Abstract

In a multicentre trial of the EORTC ECTG we have treated 43 non-pretreated patients with advanced non-small-cell lung cancer (NSCLC) with the new semisynthetic taxoid docetaxel (Taxotere). Six patients were ineligible; of the 37 eligible patients, ten had prior radiotherapy and 18 prior surgery. They received 100 mg m-2 in 1 h i.v. every 3 weeks, usually in an outpatient setting. Prophylactic steroids, antihistaminics or antiemetics were not routinely given. Two patients were not evaluable because they withdrew from the study because of a hypersensitivity reaction after the second cycle. The main toxicity was neutropenia (80% of cycles), although infections were rare (4%). One patient died from sepsis during neutropenia. Hypersensitivity reactions necessitating interruption of docetaxel (Taxotere) infusions were found in only 10% of cycles. The overall response rate was 23% with one complete response, and seven partial responses. Stable disease was found in 16 patients. The median duration of response was 36 weeks, and the median survival of all patients was 11 months. Docetaxel (Taxotere) is among the most active drugs for treatment of NSCLC.


Br. J. Cancer (1994), 70, 384-387 © Macmillan Press Ltd., 1994

### Docetaxel (Taxotere™) is active in non-small-cell lung cancer: a phase II trial of the EORTC early clinical trials group (ECTG)

T. Cerny[1], S. Kaplan[2], N. Pavlidis[3] P. Schöffski[4], R. Epelbaum[5], J. van Meerbeek[6], J. Wanders[7], H.R. Franklin[7] & S. Kaye[8] for the ECTG (Early Clinical Trials Group of the EORTC)

[1]*Institut für Medizinische Onkologie, 3010 Berne, Switzerland;* [2]*Abteilung für Onkologie, Kantonsspital Basel, 4031 Basle, Switzerland;* [3]*Medical Oncology Section, University of Ioannina, 45110 Ioannine, Greece;* [4]*Medizinische Hochschule Hannover, 3000 Hannover 61, Germany;* [5]*The Northern Israel Oncology Centre, Rambam Medical Centre, 31969 Haifa, Israel;* [6]*Department of Oncology and Pulmonology, University Hospital Antwerp, 2520 Edegem, Belgium;* [7]*EORTC-New Drug Development Office, 1081 JC Amsterdam, The Netherlands;* [8]*University of Glasgow, CRC Department of Medical Oncology, Bearsden, Glasgow G61 1BD, UK.*

**Summary** In a multicentre trial of the EORTC ECTG we have treated 43 non-pretreated patients with advanced non-small-cell lung cancer (NSCLC) with the new semisynthetic taxoid docetaxel (Taxotere™). Six patients were ineligible; of the 37 eligible patients, ten had prior radiotherapy and 18 prior surgery. They received 100 mg m$^{-2}$ in 1 h i.v. every 3 weeks, usually in an outpatient setting. Prophylactic steroids, antihistaminics or antiemetics were not routinely given. Two patients were not evaluable because they withdrew from the study because of a hypersensitivity reaction after the second cycle. The main toxicity was neutropenia (80% of cycles), although infections were rare (4%). One patient died from sepsis during neutropenia. Hypersensitivity reactions necessitating interruption of docetaxel (Taxotere) infusions were found in only 10% of cycles. The overall response rate was 23% with one complete response, and seven partial responses. Stable disease was found in 16 patients. The median duration of response was 36 weeks, and the median survival of all patients was 11 months. Docetaxel (Taxotere™) is among the most active drugs for treatment of NSCLC.

Lung cancer is now one of the leading causes of cancer death in both men and women. The revised WHO histological classification of lung tumours distinguishes four major cell types, i.e. squamous cell carcinoma, adenocarcinoma and large-cell and small-cell carcinoma. Non-small-cell lung cancer (NSCLC) is a systemic disease in most cases; only about 15–25% of unselected patients with these histological types of lung cancers have anatomically localised disease amenable to local treatment. Half of such patients die from their disease despite local therapy with curative intent. NSCLC is one of the tumour types in which single-agent chemotherapy has been most extensively tested (Cohen & Perevodchikova, 1979; Joss *et al.*, 1984; Hansen *et al.*, 1991). Data suitable for analysis indicate that, by current standards, response rates to single agents range from 5 to 20%. Detection of new and active agents for the treatment of NSCLC therefore requires the continuing evaluation of novel substances in carefully selected patients with a good prognosis and measurable disease.

In the late 1960s the National Cancer Institute's large-scale plant screening programme found that a crude extract of the bark from the Pacific yew, *Taxus brevifolia*, has activity against P388 mouse leukaemia. In 1971, Paclitaxel (taxol) was isolated and characterised as the active principle of the extract (Wani *et al.*, 1971). Docetaxel (Taxotere™) is a semisynthetic taxoid which has been synthesised from a precursor extracted from a renewable natural source, the needles of the European yew, *Taxus baccata*. It enhances microtubule assembly and inhibits the depolymerisation of tubulin. Docetaxel (Taxotere™) had a favourable activity profile when compared with taxol in animal models (Verweij *et al.*, 1994), and has therefore been selected for further testing in human clinical trials.

We have conducted a prospective multicentre phase II trial in order to determine the objective response rate and response duration of docetaxel (Taxotere™) in patients with advanced NSCLC.

Correspondence: T. Cerny, Institut für Medizinische Onkologie, Inselspital, CH-3010 Berne, Switzerland.
Received 28 February 1994; and in revised form 29 April 1994.

### Patients and methods

Patients (see Table I) were eligible for study participation if they had histologically or cytologically verified progressive NSCLC. The tumour had to be locally advanced, unresectable or metastatic. The presence of a bidimensionally measurable target lesion, a WHO performance status of $\leqslant 2$, a life expectancy of $\geqslant 12$ weeks, WBC $\geqslant 4.0 \times 10^9 l^{-1}$ and platelets $\geqslant 100 \times 10^9 l^{-1}$ as well as adequate renal and hepatic function were obligatory. Exclusion criteria were previous or concurrent chemotherapy, previous radiotherapy to a site used to assess response, concurrent malignancy, brain or leptomeningeal disease or concurrent treatment with other experimental drugs. A thoracic CT scan was mandatory at initial work-up. The sample size was determined according to the Gehan method.

Follow-up studies included weekly complete blood counts with differential, platelets and haemoglobin and 3-week blood controls including biochemistry (AST, ALT, bilirubin, LDH, serum creatinine, electrolytes, magnesium, alkaline phosphatase, calcium, protein, albumin; urine analysis only if indicated). For radiology a chest radiograph was required every 3 weeks; furthermore, radiographs and ultrasound scans of all measurable disease to assess response and thoracic CT scans (only when sole means of evaluation) were performed every two cycles. Additionally 3-weekly ECGs were required.

The toxicity was graded using the NCI common toxicity criteria (CTC).

Responses were classified according to the standard WHO criteria. Assignment to the progression category was done 6 weeks after entry into the study. When progression was observed between 3 and 6 weeks after entry into the study, the patient was considered to be undergoing 'early progression'. Progression could not be defined prior to 3 weeks (one full cycle) after entry into the study; patients removed from the study at earlier times for whatever reason were considered non-evaluable for response.

### *Drug administration*

Docetaxel (Taxotere™) was given at a dose of 100 mg m$^{-2}$ as a 1 h intravenous infusion every 3 weeks in an outpatient

**Table I** Patient characteristics (eligible patients: $n = 37$)

| | |
|---|---|
| Performance status (WHO) | |
| 0 | 15 |
| 1 | 21 |
| 2 | 1 |
| Sex | |
| Male | 28 |
| Female | 9 |
| Age (years) | |
| Median | 57 |
| Range | 38–74 |
| Histological subtype | |
| Adenocarcinoma | 16 |
| Squamous cell carcinoma | 11 |
| Large-cell and other undifferentiated NSCLC | 10 |
| Pretreatment | |
| Surgery | 18 |
| Radiotherapy (18–56 Gy) | 10 |
| Tamoxifene | 1 |

setting. Docetaxel (Taxotere™) was diluted with 5% dextrose or 0.9% saline to yield an intermediate solution of 10 mg $ml^{-1}$. This solution was immediately shaken for 20 s using a mixer in order to obtain a clear solution. The appropriate amount of the drug to be administered was further diluted in 5% dextrose or 0.9% saline to produce a maximum concentration of 1 mg $ml^{-1}$. The dose administered was reduced to 75 mg $m^{-2}$ in patients with haematological or other toxicities. Reducing the dose by 25% of the previous dose was necessary in case of a neutrophil nadir of $< 0.5 \times 10^9 l^{-1}$ lasting more than 7 days and/or with fever ($\geqslant$ 38.5°C) requiring i.v. antibiotics. The same reduction was done in case of a platelet nadir of $< 25 \times 10^9 l^{-1}$. A treatment delay of 1 week was mandatory if on day 22 the neutrophil nadir was $< 1.5 \times 10^9 l^{-1}$. If no recovery was achieved after 1 week, the patient was withdrawn from the study.

Prophylactic steroids, antihistamines and antiemetics were prohibited for the first cycle.

Guidelines for modification of the administration of docetaxel in the cases of hypersensitivity reactions were as follows. For mild symptoms, i.e. localised cutaneous reaction, pruritus or flushing, the rate of infusion was to be decreased until disappearance of symptoms. Docetaxel infusion was, thereafter, completed. With moderate symptoms, such as generalised pruritus, dyspnoea or hypotension (blood pressure still above 80 mmHg), the infusion of docetaxel was to be halted and antihistamines and i.v. corticosteroids were to be given. The docetaxel infusion was to be resumed after disappearance of symptoms. Premedication with corticosteroids and antihistamines was then optional in the subsequent course of treatment. Bronchospasms, generalised urticaria, hypotension with a systolic blood pressure of less than 80 mmHg and angio-oedema were considered severe symptoms, and if these occurred the docetaxel infusion was stopped and antihistamines and steroids given. If possible, the docetaxel infusion was resumed within 3 h after the patient recovered and in this case premedication was mandatory. Premedication was also necessary with subsequent cycles and comprised dexchlorpheniramine i.v. 5–10 mg and orally 5 mg and dexamethasone 5–10 mg i.v. and orally 20 mg, within 1 h before treatment.

In the case of grade 1 cutaneous reactions, according the NCI common toxicity criteria, no dose modification resulted. If grade 2 cutaneous reactions were observed, the subsequent dose was reduced by 25%; in the case of grade 3 toxicity, retreatment was delayed 1 week and then a dose modification had to be applied.

### Results

Forty-three patients were enrolled in the trial between May and August 1992, 37 being eligible and evaluable for analysis. Reasons for non-eligibility were: non-measurable lesions (three patients), SCLC (one patient) and unstable cardiovascular disease (one patient). In addition one patient was never treated and had pre-existing significant neuropathy. There were 28 male and nine female patients with a median age of 57 (38–74) years. Of the 37 eligible patients 15 had a WHO performance status of 0, 21 were WHO grade 1 and one was WHO grade 2. Prior surgery was performed in 18 patients and prior radiotherapy in 10. One patient was pretreated with tamoxifen. The histological subtype of NSCLC was adenocarcinoma in 16 patients, squamous cell carcinoma in 11 patients, large-cell carcinoma in four patients and large-cell undifferentiated or other undifferentiated NSCLC in ten patients.

In total 167 cycles were administered (1–11 cycles per patient); the median cumulative dose given was 325 mg $m^{-2}$ per patient (range 100–1016 mg $m^{-2}$). The median dose intensity (mg $m^{-2}$ per week) was 29.9 (range 16.3–33.5). Dose reductions were necessary in 46 (27%) of the 167 cycles: in 15 (9%) because of haematological toxicity, in 25 (15%) because of non-haematological toxicity and in six (4%) for both reasons. Treatment delays were recorded in 13 (8%) of the cycles; in six (4%) this was drug related. Treatment interruptions due to hypersensitivity were necessary in 16 (10%) of the 167 cycles.

Reasons for withdrawal from the study were disease progression in 18 patients, toxicity in seven (fluid retention in two, hypersensitivity in two, asthenia in one, paraesthesia and arthralgia in one, venous thrombosis at the injection site in one), patient refusal in three, end of protocol in six, intercurrent death in five.

### *Haematological toxicity (see Table II)*

Significant leucopenia was found in 87% of the cycles and neutropenia in 95% of the cycles, being CTC grade 3 and 4 in 62%. Febrile neutropenia occurred in nine cycles (5%); proven infections were only seen in 4% of the cycles, while on patient died from a neutropenia-related septicaemia. Thrombocytopenia and anaemia, when they occurred, were only mild.

### *Non-haematological toxicity (see Tables III and IV)*

Complete hair loss occurred universally in all patients receiving two or more cycles. Skin toxicity was also common (65%), but usually of mild to moderate severity. Symptoms included pruritus, dry skin, erythema and desquamation. Furthermore, nail changes consisting of calcification and/or onycholysis were found in 22% of patients. Asthenia, malaise and fatigue were encountered in 57% of the patients, and diarrhoea in 13% of cycles. Weight gain, oedema and pleural

**Table II** Drug-related haematological toxicity per cycle ($n = 167$)

| *Common toxicity criteria* | *I* | *II* | *III* | *IV* | *Total* | *%* |
|---|---|---|---|---|---|---|
| Leucopenia | 24 | 56 | 57 | 9 | 146 | 87 |
| Neutropenia | 8 | 22 | 50 | 54 | 134 | 80 |
| Anaemia | 68 | 9 | 1 | 0 | 78 | 47 |
| Thrombocytopenia | 15 | 0 | 0 | 0 | 15 | 9 |

**Table III** Drug-related non-haematological toxicity per patient ($n = 37$)

| *Common toxicity criteria* | *I* | *II* | *III* | *IV* | *NK*[a] | *Total* | *%* |
|---|---|---|---|---|---|---|---|
| Alopecia | 5 | 29 | 1 | – | – | 35 | 95 |
| Skin toxicity | 8 | 11 | 2 | 1 | 2 | 24 | 65 |
| Asthenia/malaise/fatigue | 9 | 8 | 4 | 0 | 0 | 21 | 57 |
| Neurological | 11 | 3 | 0 | 0 | 0 | 14 | 38 |
| Oedema | 5 | 5 | 1 | 0 | 1 | 12 | 32 |
| Pleural effusion | 3 | 2 | 1 | 0 | 1 | 7 | 19 |
| Weight gain | 3 | 1 | 0 | 0 | 0 | 4 | 11 |
| Nail changes/onycholysis | 6 | 2[b] | 0 | 0 | 0 | 8 | 22 |

[a]Grading not known. [b]Onycholysis.

**Table IV** Most frequent drug-related non-haematological toxicity per cycle ($n = 167$)

| *Common toxicity criteria* | *I* | *II* | *III* | *IV* | *NK*[a] | *Total* | *%* |
|---|---|---|---|---|---|---|---|
| Pain | 14 | 10 | 4 | 0 | 0 | 28 | 17 |
| Nausea | 12 | 8 | 3 | 0 | 0 | 23 | 14 |
| Diarrhoea | 9 | 10 | 1 | 1 | 0 | 21 | 13 |
| Fever | 6 | 10 | 0 | 0 | 1 | 17 | 10 |
| Allergy (hypersensitivity response) | 7 | 6 | 4 | 0 | 0 | 17 | 10 |

[a]Grading not known.

effusions were documented in 11%, 32% and 19% respectively, the first symptoms occurring after a median cumulative dose of $400 \text{ mg m}^{-2}$. Allergy (hypersensitivity) was seen in 30% of the patients and 10% of the cycles. It was grade 3 in 11% of the patients but no grade 4 hypersensitivity was seen. Mild fever was seen in 27%, vomiting in 16%, stomatitis in 13% and myalgia or headache in 11%. Local phlebitis was found in 8%.

Neurological side-effects (38%), usually sensory neurotoxicity (35%), were mostly mild and the symptoms are shown in Table V. Pulmonary symptoms occurred in 30% of the patients, but they were related to fluid retention or hypersensitivity. CTC grade 3 and 4 pulmonary symptoms were found in 8% of patients. Other toxicities only occurred infrequently.

### *Responses (see Table VI)*

Two eligible patients did not receive two full cycles of treatment because of hypersensitivity reactions, and were therefore considered inevaluable for response. Of 35 patients evaluable for response there was one complete remission (3%) lasting 5 months. At that time adjuvant radiotherapy was given, after which the patient has an ongoing response for 14 months. Partial responses were seen in seven patients (20%) and stable disease was found in 16 patients (46%). All objective responses were subject to independent external review. Eleven patients (31%) progressed during treatment. Duration of response (complete, partial and no change) and duration of survival were calculated from the commencement of treatment. Patients were censored for duration of response and time to progression at the date of further treatment. The responses lasted 15–48 weeks with a median duration of response according to the Kaplan–Meier method of 36 weeks (Figure 1). According to the same method the median survival of evaluable patients was 11 months.

**Table V** Specifications of neurological toxicity per cycle ($n = 167$)

| *Common toxicity criteria* | *I* | *II* | *III* | *IV* | *Total* | *%* |
|---|---|---|---|---|---|---|
| Sensory | 33 | 4 | 0 | 0 | 37 | 22 |
| Motor | 3 | 1 | 0 | 0 | 4 | 2 |
| Cortical | 1 | 0 | 0 | 0 | 1 | <1 |
| Other (forehead numbness) | 1 | 3 | 0 | 0 | 4 | 2 |

**Table VI** Evaluation of response (WHO classification) ($n = 37$)

| *Response* | *n* | *%* |
|---|---|---|
| Complete remission | 1 | 3 |
| Partial remission | 7 | 20 |
| No change | 16 | 49 |
| Progression | 11 | 29 |
| Not evaluable | 2 | |

**Figure 1** Duration of response, plotted according to the method of Kaplan and Meier.

### Discussion

No widely tested single agent has a response rate of more than 30% in NSCLC. Responses that do occur after single-drug therapy are brief; complete remissions are rare. Ifosfamide, vinblastine, etoposide, mitomycin C, cisplatin, vindesine, vinorelbine and edatrexate are generally regarded as being the most active agents. Several studies have shown higher response rates to combination chemotherapy compared with single agents. However, the combinations are not necessarily associated with improved survival compared with single agents, but they are universally associated with increased toxicity. With currently available drug combinations, the impact on survival in metastatic disease is to be regarded as being modest. New agents and drug combinations are needed to improve the prognosis of advanced NSCLC. Docetaxel (Taxotere™) is a new promising drug for the treatment of malignant diseases. The analysis of this phase II multicentre trial in patients in good clinical condition with advanced, non-resectable or metastatic NSCLC reveals an impressive activity of this novel taxoid. Docetaxel (Taxotere™), when given as a single drug at a dose of $100 \text{ mg m}^{-2}$ every 3 weeks on an outpatient basis, is generally well tolerated. The dose-limiting toxicity in this schedule is short-lasting leucopenia/neutropenia, rarely being associated with infections. Other common toxicities are alopecia, skin toxicity, fatigue, nausea, neurological toxicity, diarrhoea, pain and hypersensitivity reactions. Fluid retention, associated with oedema, weight gain and pleural effusions, is another common observation in patients treated with the agent at this dose and schedule. One potentially treatment-related death occurred in this series due to septicaemia during the second cycle. Another patient died due to *Candida tropicalis* pneumonia, after an episode of neutropenia had completely resolved.

In a similar study, investigators at the Memorial Sloan-Kettering Cancer Center reported a preliminary response rate with docetaxel (Taxotere™) of 28% in 18 evaluable patients with NSCLC (Rigas *et al.*, 1993). Paclitaxel (Taxol) has also been found to be active in non-small-cell lung cancer with a response rate of 22% (Donehower & Rowinsky, 1993).

Further follow-up is needed to determine the definitive response duration of this active and generally well-tolerated new drug. The impact of concomitant steroids on the drug profile of docetaxel (Taxotere™) needs further evaluation. The combination of docetaxel (Taxotere™) with other active agents for treatment of NSCLC, such as cisplatin or ifosfamide, is presently under investigation.

### References

COHEN, M.H. & PEREVODCHIKOVA, N.I. (1979) Single agent chemotherapy of lung cancer. In *Lung Cancer: Progress in therapeutic Research*, Muggia, F.M., Rozencweig, M. (eds). pp. 343–374. Raven Press: New York.

DONHOWER, R.C. & ROWINKSY, E.K. (1993). An overview of experience with Taxol (Paclitaxel) in the USA. *Cancer Treat Rev.*, **19** (Suppl. C), 63–78.

HANSEN, H.H., RORTH, M., PINEDO, H.M., LONGO, D.L. & CHABNER, B.A. (eds). (1991). *Cancer Chemotherapy and Biological Response Modifiers*. Annual 12, pp. 449–459. Elsevier Scientific Publishers: Amsterdam.

JOSS, R.A., CAVALLI, F. & GOLDHIRSCH, A. (1984). New agents in non-small cell lung cancer. *Cancer Treat. Rev.*, **11**, 205–236.

RIGAS, J.R., FRANCIS, P.A., KRIS, M.G., PISTERS, K.M.W., KURIE, J., WOOLEY, K.J., KLEMM, K.L., HANNA, M.T. & HEELAN, R.T. (1993). Phase II trial of Taxotere in non-small cell lung cancer. *Proc. Am. Soc. Clin. Oncol.*, **12**, 336.

TOMIAK, E., PICCART, M.J., KERGER, J., DEVALERIOLA, D., TUENI, E., LOSSIGNOL, D., LIPS, S., LE BAIL, N. & BAYSSAS, M. (1991). A phase I study of Taxotere (RP 56976, NSC 628503) administered as a one hour intravenous (i.v.) infusion on a weekly basis. *Eur. J. Cancer*, **27** (Suppl. 2), 1184.

VERWEIJ, J., CLAVEL, M. & CHEVALIER, B. (1994). Paclitaxel (Taxol) and docetaxel (Taxotere): not simply two of a kind. *Ann. Oncol.* (in press).

WANI, M.C., TAYLOR, H.L., WALL, M.E., COGGON, P. & MCPHAIL, A.T. (1971). Plan antitumor agents VI. The isolation and structure of taxol, a novel antileukemic and antitumor agent from *Taxus brevifolia*. *J. Am. Chem. Soc.*, **93**, 2325–2327.


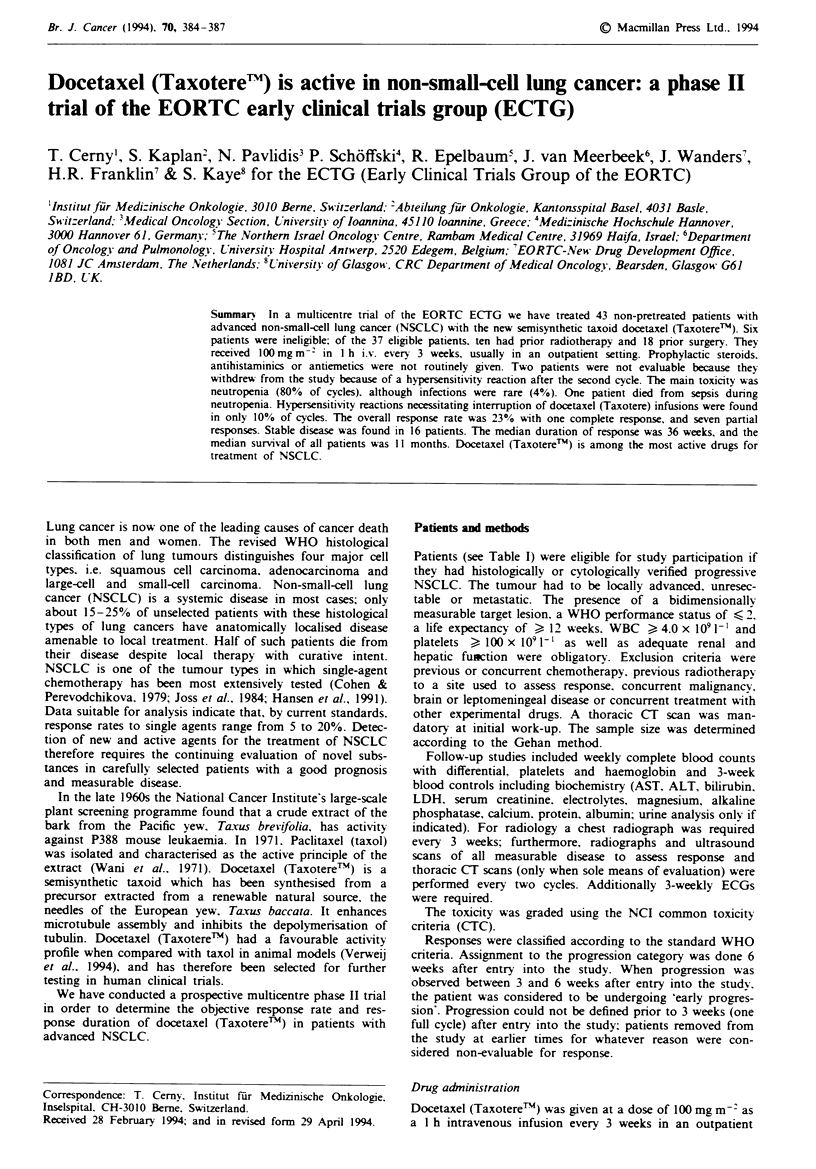

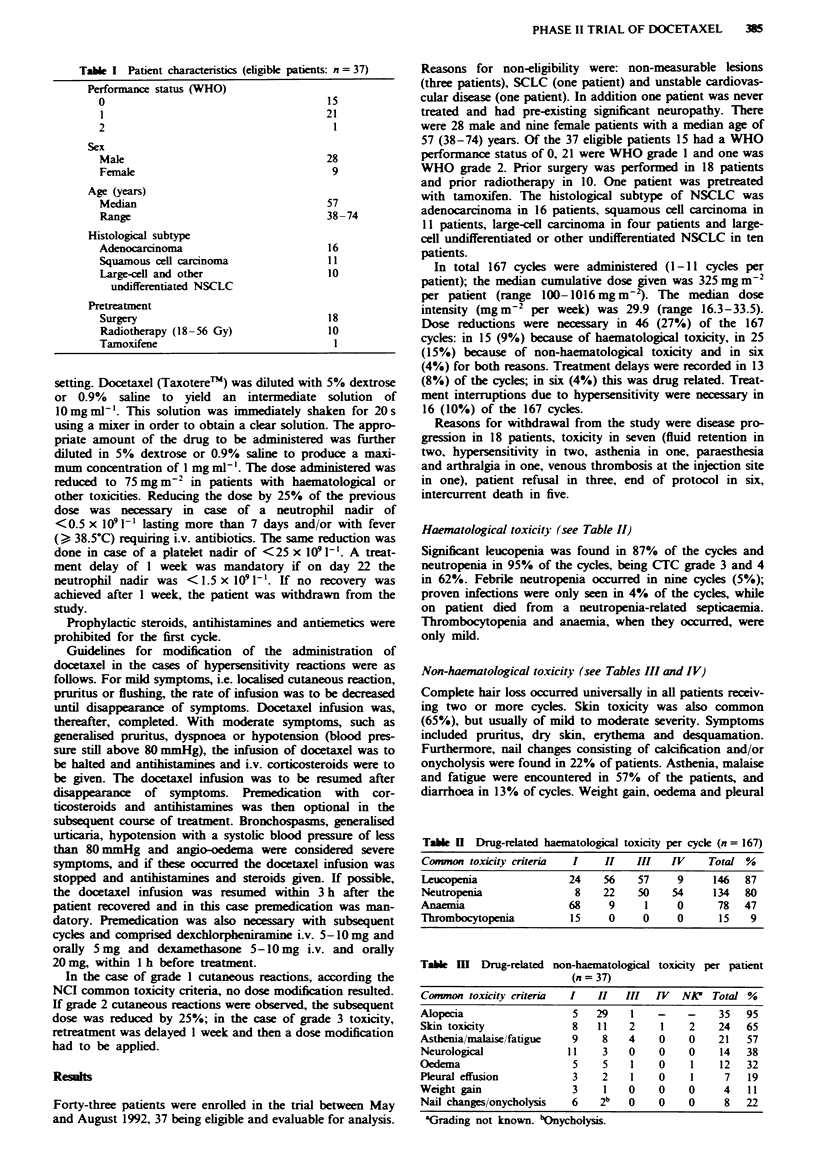

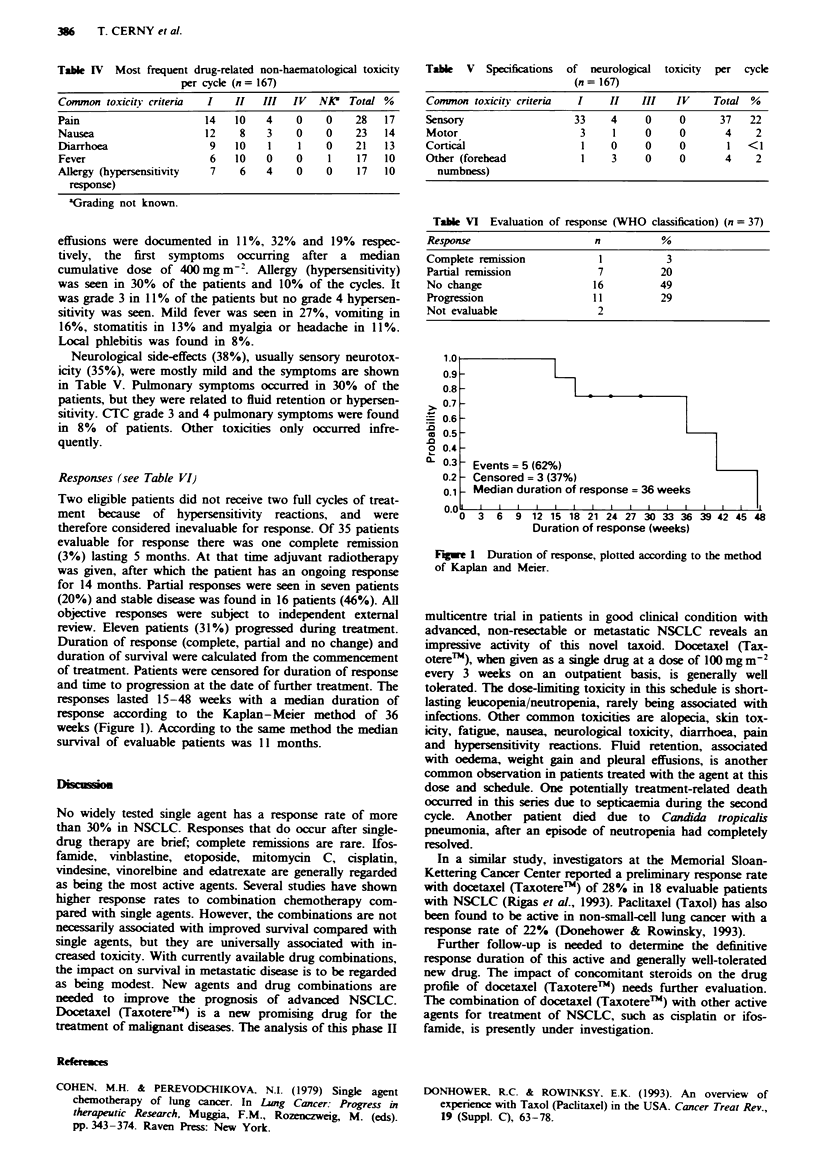

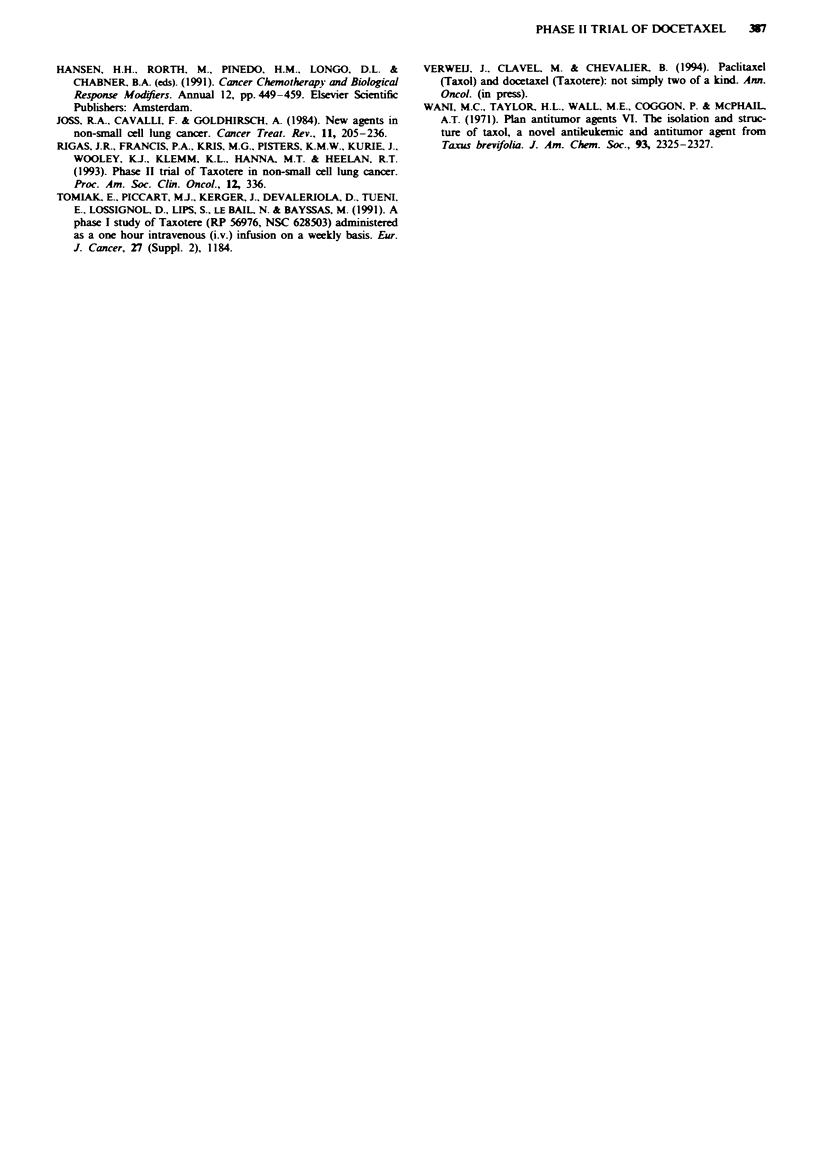


## References

[OCR_00490] Hansen H. H., Rørth M. (1991). Lung cancer.. Cancer Chemother Biol Response Modif.

[OCR_00498] Joss R. A., Cavalli F., Goldhirsch A., Mermillod B., Brunner K. W. (1984). New agents in non-small cell lung cancer.. Cancer Treat Rev.

[OCR_00518] Wani M. C., Taylor H. L., Wall M. E., Coggon P., McPhail A. T. (1971). Plant antitumor agents. VI. The isolation and structure of taxol, a novel antileukemic and antitumor agent from Taxus brevifolia.. J Am Chem Soc.

